# Comparison between continued inpatient treatment versus day patient treatment after short inpatient care in early onset anorexia nervosa (COTIDEA trial): a study protocol for a non-inferiority randomised controlled trial

**DOI:** 10.1186/s12888-023-05222-9

**Published:** 2023-10-10

**Authors:** A. Ayrolles, A. Bargiacchi, J. Clarke, M. Michel, F. Baillin, V. Trebossen, H. Poncet Kalifa, S. Guilmin-Crépon, R. Delorme, N. Godart, C. Stordeur

**Affiliations:** 1 Child and Adolescent Psychiatry Department, Reference Center for Rare Early-Onset Restrictive Eating Disorder, Robert Debré University Hospital, APHP, Paris, France; 2 Université Paris Cité, Paris, France; 3 Human Genetics & Cognitive Functions, CNRS UMR3571, Institut Pasteur, Paris, France; 4 Institute of Psychiatry and Neuroscience of Paris, INSERM U1266, Paris, France; 5 CMME (GHU Paris Psychiatrie Et Neurosciences), Paris Descartes University, Paris, France; 6 Inserm, ECEVE, U1123, 10 Boulevard de Verdun, 75010 Paris, France; 7 Department of Clinical Epidemiology, Robert Debré University Hospital, APHP, Paris, France; 8 Pediatric Endocrinology-Diabetology Department, Reference Center for Rare Growth and Development Endocrine Diseases, INSERM NeuroDiderot, Robert Debré University Hospital, APHP, Paris, France; 9 CESP, INSERM, UMR 1018, University Paris-Sud, UVSQ, University Paris-Saclay, Villejuif, France; 10 UFR Simone Veil, UVSQ, University Paris-Saclay, Montigny-Le-Bretonneux, France; 11 Fondation de Santé Des Etudiants de France, Paris, France

**Keywords:** Early-onset anorexia nervosa, Day-treatment, Cost-effectiveness

## Abstract

**Background:**

In children with early-onset anorexia nervosa (first symptoms before 13 years old, EO-AN), experts recommend initial outpatient treatment but in-patient treatment (IP) is frequently indicated due to acute medical instability or for those who have not improved with outpatient treatment. This IP can target either a partial weight restauration or a total weight normalization (return to the previous BMI growth trajectory). There are no evidence in the literature on which is the better therapeutic option in EOAN. But as long length of stay induce social isolation, with elevated costs, we wonder if a stepped-care model of daypatient treatment (DP) after short IP stabilisation may be a treatment option as effective as full-time IP to target weight normalization. We designed a two-arm randomised controlled trial testing the non-inferiority of a stepped-care model of DP after short IP stabilisation versus full-time IP.

**Methods:**

Eighty-eight children aged 8 to 13 years suffering from EOAN with initial severe undernutrition will be randomly allocated to either IP treatment as usual or a stepped care DP model both targeting weight normalization. Assessments will be conducted at inclusion, somatic stabilization, weight normalization, 6 months and 12 months post randomisation. The primary outcome will be BMI at 12 months post-randomisation. Secondaries outcomes will included clinical (tanner stage), biological (prealbumin, leptin, total ghrelin and IGF1) and radiological (bone mineralization and maturation) outcomes*,* eating symptomatology and psychiatric assessments, motivation to change, treatment acceptability and quality of life assessments, cost-utility and cost-effectiveness analyses.

**Discussion:**

COTIDEA will provide rigorous evaluation of treatment alternative to full-time inpatient treatment to allow a reduction of social iatrogenic link to hospital length of stay and associated costs.

**Trial registration:**

Trial is registered on ClinicalTrials.gov (NCT04479683).

## Background

Early-onset anorexia nervosa (EOAN) is characterised by disease onset in children before the age of 13 years old and will thus include prepubertal and post pubertal onset [1]. Compared to the classic adolescent form of anorexia nervosa (AN), EOAN presents clinical and epidemiological specificities [1]. Children with EOAN more often present severe restrictive forms with more rapid weight loss, associated with a greater frequency of total aphagia and the presence of associated "non-specific" somatic symptoms (digestive complaints, abdominal pain) [2–4]. Frequently requiring inpatient treatment, children with EOAN also have a higher average length of stay (LOS) than adolescents with AN [1]. Despite an increased prevalence [5] and a specific burden in pediatrics and mental health departments, treatment knowledge remains sparse, and based on mainstream clinical opinion with little empirical standing [6]. In clinical practice, in children with AN, whereas experts recommend outpatient treatment (OP), in-patient treatment (IP) remains frequently indicated due to acute medical instability [6] or for those who have not improved with OP. Long LOS, social isolation, and elevated costs have led to discuss different treatment options with age-appropriate strategies for child population [6, 7].

In recent years, in adolescents with a mean age of 15 years old, day patient treatment (DP) following initial IP has been developed [8, 9]. Results support comparable efficacy, better acceptability and lower cost in the management of moderate AN, compared to prolonged IP, but also better social adjustment and more age-adequate autonomy [6, 8]. A Cochrane meta-analysis reports no difference between specialised full-time IP compared to combined brief IP and OP in weight gain at 12 months follow-up [10]. Similar clinical and cost effectiveness evaluations are currently conducted in adult AN population to compare IP to combined IP/DP [11]. Rigorous assessment of this treatments approaches is necessary to adapt national and international guidelines. Particularly, replication of randomised controlled trial focusing on younger populations under 13 years old is needed to test alternative care modalities to extended IP. In children aged 8–13 years with EOAN, somatic condition frequently requires continuous inpatient monitoring [1], DP cannot initially be proposed. We wonder if we could shorten the duration of IP by relaying care in a one day a week DP after somatic stabilisation, with a comparable effectiveness in weight gain and maintenance, a better acceptability, a better evolution in terms of school and friendship integration, and a lower cost.

COTIDEA trial aims to compare a stepped-care model of DP after short IP stabilisation to full-time IP in a two-arm open-label, single centre, non-inferiority randomised controlled trial in children and adolescents under 14 years old with EOAN and initial severe undernutrition.

The specific objectives of the propose study are to:

1. Demonstrate non-inferiority of a stepped-care model of DP after short IP stabilisation versus full-time IP based on BMI at 12 months post-randomisation (primary outcome).
2. Demonstrate non-inferiority of a stepped-care model of DP after short IP stabilisation in term of clinical, biological outcomes, AN symptoms, comorbidities, and relapse at different time points (weight normalization, 6, 12 months).
3. Assess the impact of a stepped-care model of DP after short IP stabilisation versus full-time IP on treatment satisfaction and quality of life of patients and parents at 6 and 12 months.
4. Assess the impact of the prospect of discharge to DP on initial IP (duration before somatic stabilisation, motivation to change) at inclusion and somatic stabilisation.
5. Determine factors associated with positive response to stepped-care model of DP after short IP.
6. Determine the cost-utility and cost-effectiveness of a stepped-care model of DP after short IP stabilisation compared with full-time IP.

## Methods

### Study design and participants

The study is a randomised, single-centre, controlled, non-inferiority trial conducted in the Child and Adolescent Psychiatry Department of the Robert Debre University Hospital in France (protocol version n°1.2 02182020). This unit provide specialised treatment for children and adolescents with EOAN. The study protocol was approved by local ethics committee (ID-RCB: 2019–102408-49, CPP: 19.09.12.56704). Trial is also registered on ClinicalTrials.gov (NCT04479683).

Participants will be aged from 8 to 13 years inclusive at inclusion with diagnosis of anorexia nervosa according to DSM 5 criteria [12], requiring full time IP according to the French guidelines [13, 14] and affiliated to the French social security system. Only participants with no prior hospitalisation in a specialised eating disorder care unit will be eligible after informed consent of the holder(s) of parental authority.

Patients with other eating disorder (Avoidance/Restrictive Food intake Disorder or Eating disorders not otherwise specified according to DSM 5 criteria [12]), underlying unbalanced somatic diseases (in particular chronic inflammatory bowel disease, dysthyroidism, diabetes, adrenal insufficiency, diabetes insipidus, hemopathies, brain tumours, tuberculosis) will be excluded. We will also exclude children with contraindication to outpatient care due to indication of maintain IP for other reasons according to French guidelines (either with a psychiatric indication for continued hospitalisation such as severe suicidal ideations or with environmental indications for continued full-time hospitalisation (absence of family to accompany ambulatory care or impossibility due to distance)) [13].

### Procedure (Fig. 1)

**Fig. 1:**
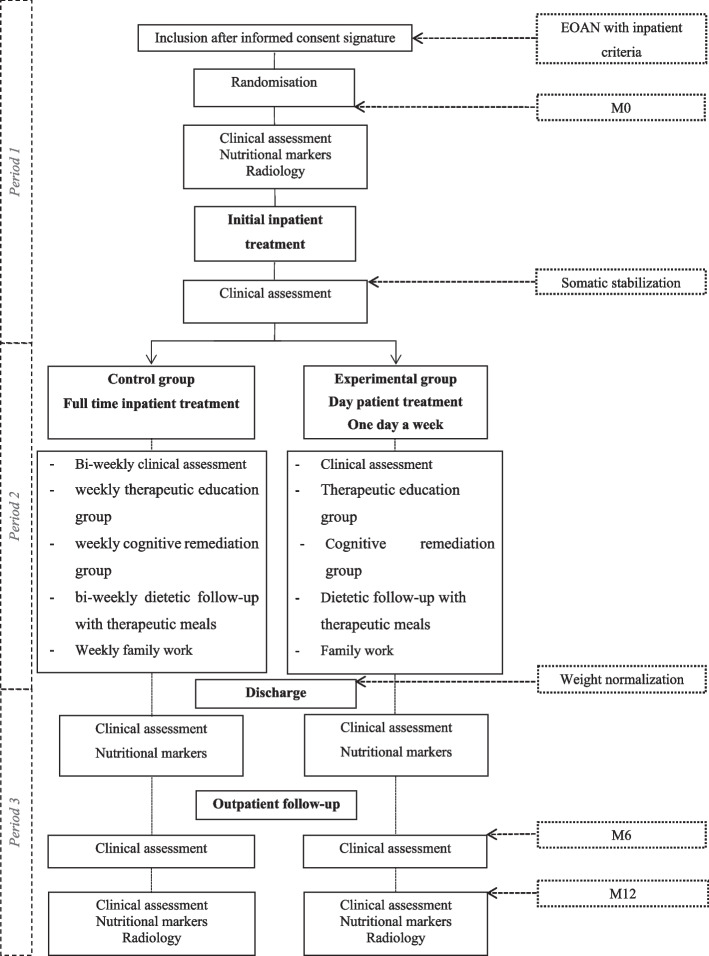
Study flowchart

At initial clinical assessment, eligible children and caregivers will be informed of the study and study protocol is fully explained by the investigator. After signature of the informed consent by the holder(s) of parental authority, target weight for weight normalisation will be calculated and participants will be randomised into one of the two trial arms. Weight normalisation is individualised and will be determined at inclusion and before randomisation to return to the previous BMI growth trajectory and is calculated by two different investigators [15–18].

Randomisation will be done by a randomisation list with the nQuery Advisor and nTerim 4.0 software implemented in the eCRF. Children and parents are informed of the result of the randomisation by the investigator.

After inclusion and randomisation, participants will be managed in 3 phases:

*period 1: *Full-time IP until somatic stabilisation (see below for definition); *period 2:* DP or IP according to their randomisation arm until weight normalisation is reached; *period 3:* OP follow-up.

*Period 1: *Full-time IP—identical for all included children- ongoing until somatic stabilisation, which is defined as: BMI over the 3^rd^ percentile, disappearance of somatic severity criteria for undernutrition such as bradycardia (heart rate below 40 per minute during sleep time or below 50 per minutes during day time), persistent disturbances of the biological balance (hypoglycaemia, hypophosphoremia, haemolysis, cytolytic hepatitis, elevated creatine kinase), or any somatic or biological manifestation requiring constant surveillance in hospital with usual medical, dietary and psychological care (persistent refusal to drink or eat, lipothemia, hypothermia). The treatment provides multi-disciplinary care for the child and his/her family as described in French guidelines [14], including:

- Child follow-up of AN and psychiatric comorbidities by a senior psychiatrist on a bi-weekly basis
- Endocrinologic and somatic evaluation by a senior endocrinologic paediatric for initial assessment M6 and M12 and by a psychiatrist resident for follow-up during IP and by a general practitioner during OP
- Nutritional monitoring with a bi-weekly dietetic follow-up
- Psychological follow-up with weekly family therapy
- Therapeutic education for the child with a weekly psychoeducation group and day-care nurse follow-up

We will target a rapid weight gain from 500g to 1.5kg a week depending on the tolerance [19].

*Period 2: *At the end of the first period, patients start to receive treatment according to their randomisation arm until the weight normalization is reached. *For the control group*, full-time IP is continued as described in period 1 with additional cognitive remediation weekly group and weekly individual cognitive-based therapy until the weight normalisation is reached. During this time period, IP patients can benefit from permission to go home twice a week on Wednesday afternoons and weekends. *For the experimental group*, participants are discharged from IP and switch to DP one day per week until weight normalisation is reached.

The standardized DP treatment combines a medical assessment by a senior psychiatrist, family oriented treatment (parents' group and multi-family therapy session), a psychoeducation group, a cognitive remediation group, a dietetic follow-up with therapeutic meals and body constants monitoring (heart rate and blood pressure). Patients in the experimental group can return to their school activities and home life. During this period, the number of individual cognitive-based therapy sessions out from the hospital will be reported. If absence of weight gain leading to a BMI < 3rd percentile or weight loss of more than 10% of body weight during DP, patient will be readmitted to full-time IP until the weight normalisation is reached.

*Period 3: *After weight normalisation is reached, all patients will be provided the same OP follow-up until 12 month with a psychiatric consultation every month for 3 months and then once every 2/3 months as proposed in usual care.

Clinical, biological and psychometric assessments are done at baseline, at somatic stabilisation, at reaching weight normalisation, at 6 months and 12 months following study inclusion.

### Measures (Table 1)

**Table 1:** Overview of measures and time of assessment

Assessment	Inclusion	Stabilisation	Weight normalization	6 months	12 months
*Clinical assessment weight, height, BMI *	x	x	x	x	x
tanner stage	x			x	x
*Nutritional markers* prealbumin, leptin, total ghrelin and IGF1	x		x		x
*Radiology* Bone mineralization (dual-energy X-ray absorptiometry) Bone maturation (en face radiograph of the left wrist)	x				x
*Global psychosocial* CGI	x		x	x	x
*Eating symptomatology* EDI-c, MRS	x		x	x	x
*Anxiety and depressive symptomatology* CDI, STAIC	x		x	x	x
*Self-esteem Scale* Rosenberg	x		x	x	x
*Motivation to change* HAQ-11S, Motivation questionnaire	x		x	x	x
*Treatment acceptability and quality of life* CSQ-8, SF-12	x		x	x	x
Resource consumption		x	x	x	x

#### Primary outcome

The primary non-inferiority outcome is BMI (Weight in kg/(Height in m^2^)) at 12 months after inclusion to assess non-inferiority on BMI maintenance.

#### Secondary outcomes

*Biological and radiological outcomes* will be collected. *Nutritional status* will be assessed with prealbumin, leptin, total ghrelin and IGF1. Body composition and *bone mineralisation* will be estimated by using dual-energy X-ray absorptiometry (DEXA, GE Lunar ProdigyCorp., Madison. WI). Bone mineral measurements will be expressed as standard deviation score according to age and sex (SDS). *Bone maturation* will be assessed from an en face radiograph of the left wrist, interpreted using the Greulich and Pyle atlas.

*Global clinical and psychological evaluation* will be assessed with the *Clinical Global Impression* (CGI) [20] scale. CGI is scored on a two-part 7 response levels evaluating illness severity (CGI-S) and improvement (CGI-I) from 1 (“not ill”/ “very much improved”) to 7 (“extremely ill”/ “much worse”).

*Eating disorder symptomatology* will be assessed with the *Morgan and Russell scale* (MRS) [21] and the *Children Eating Disorders Inventory* (EDI-c) [22, 23]. The MRS is a questionnaire designed to evaluate the long-term evolution of the eating disorder and is used to assess the main aspects of anorexia nervosa over a six-month period. A version has been adapted for younger children, with deletion of non-appropriated items as "menstruation", "psychosexual functioning" and "emancipation". The assessment of the adapted procedure has 8 subscales divided into 3 groups: Eating; Mental status; Socioeconomic Status.

The EDI-c is adapted form of the EDI for children from 8 years of age onwards [22, 23]. This is a multidimensional self-questionnaire that assesses different psychological, behavioural and emotional characteristics associated with eating disorder symptoms. It comprises 91 items divided into 11 factors: Drive for Thinness, Bulimia, Body Dissatisfaction, Low Self-Esteem, Personal Alienation, Interpersonal Insecurity, Interpersonal Alienation, Interoceptive Deficits, Emotional Dysregulation, Perfectionism, Asceticism, and Maturity Fears. Each of the 91 items is rated from 'never' to 'always', respectively from 0 to 3 for direct items and from 3 to 0 for indirect items.

*Depressive and anxiety symptomatology* will be respectively assessed by the Children Depression Inventory (CDI) [24], the *State –Trait Anxiety Inventory for Children* (STAIC) [25]. The CDI is a self-adapted Beck Depression Inventory for children aged 7 to 17 years, with 27 items scored from 0 (absent or normal behaviour for age) to 2 (severe). The total score, calculated as the sum of all the items, varies from 0 to 54 with a pathological threshold of 15. It is a widely used tool in the international literature to assess the intensity of depression.

The STAIC is a self-questionnaire and comprises two 20-item subscales, the Trait Anxiety and the State Anxiety. The scores observed for the two subscales range from 20 to 80, with a score of 35 or less corresponding to very low anxiety and a score of over 65 corresponding to very high anxiety.

*Self-esteem will be assess by the Rosenberg Self-esteem Scale * [26]* is* a self-questionnaire with a 10-items measure of global self-esteem, widely used in general and clinical populations. The higher the scores, the better the self-esteem. It can be used from the age of 8 years.

*Therapeutic alliance/perception of treatment benefit and Motivation to change* will be assessed by the *Helping Alliance Questionnaire-11* (HAQ-11S) [27], the *Consumer Satisfaction Questionnaire* (CSQ-8) [27] and the *Motivation Questionnaire* [28].

The CSQ-8 is a self -"satisfaction"-questionnaire, defined here as a concept tending to assess whether the needs of the consumer of a service are being met or not. In medical terms, patient satisfaction is an internationally recognized measure for the evaluation of the perception of quality of care; this dimension can have an influence on the outcome and adherence to the proposed treatment and thus on the acceptability of the treatment. It consists of eight questions, each with four response options ranging from one "not at all satisfied" to four "completely satisfied".

The HAQ-11S is a self-questionnaire of 11 items measuring the strength of the collaboration between the patient and the healthcare team.

The Motivation Questionnaire will assess the importance of change and the perceived ability to change. It consists of two questions: "How important is it for you to change" and "How confident are you in your ability to change". Both questions are rated on a Likert scale from 0: "Not at all" to 10: "Completely".

*Quality of life* will be assessed by the *Short-Form 12-Item Health Survey* (SF-12) [29, 30]. The SF-12 is a generic self-assessment scale of quality of life and a shortened version of the SF-36 validated in France. It contains 12 questions. It provides two scores: a physical health and a mental health summary scores. These two scores were constructed so that their average in the general population is 50 and range from 0 to 100, 100 indicating the highest level of quality of life.

*Social functioning* will be particularly evaluated based on the subscales of the MRS assessing the socioeconomic status: relationship with family, social contacts outside family, social activities and scolarity.

*Direct and indirect costs* will be calculated from resources consumption: all hospital admissions will be recorded as ambulatory consultations, complementary examinations, medical transport and number of non-working days for parents due to medical care for their child for control and experimental groups will be recorded.

### Data collection and management

A checklist of the measures performed at each visit will be completed by the investigator and assessment visits will be scheduled as soon as the patient is discharged from hospital to promote participant retention and complete follow-up. Collected data will be implemented in the eCRF, data will be managed and processed by the clinical epidemiology unit at Robert Debré Hospital with independence from the sponsor. The biological samples collected for the determination of leptin and ghrelin will be stored in a biological collection, they will be kept in the Biochemistry and Hormonology Laboratory at Robert Debré hospital for the duration of the study. At the end of the research and after dosage, the samples will be destroyed. The AP-HP (Assistance Publique – Hôpitaux de Paris) is the sponsor and owner of the data and it may not be used or transmitted to a third party without its prior agreement. Audits may be conducted at any time by persons appointed by the sponsor and independent of the investigators. Its purpose is to ensure the quality of the research, the validity of its results and compliance with the law and regulations currently applicable. The persons responsible for the quality control of research involving the human person (article L.1121–3 of the French Public Health Code) will take all necessary precautions to ensure the confidentiality of information relating to the research, to the persons taking part in it and in particular to their identity and to the results obtained.

### Data analysis

#### Sample size

We calculated the sample size for a one-sided Student’s t test with a significance level of 2.5% and a power of 90% based on the clinically determined non-inferiority margin for a difference in BMI of 1 kg/m2, assuming a distribution of the BMI of 16,6 ± 1,35 kg/m^2^. This procedure led to a required sample size of 80 patients (40 per group). Assuming a dropout rate of 10% [8], we calculated a sample size of 88 patients. We aim to include 88 children with EOAN (44 per group).

#### Statistical analysis plan

The statistical analysis of the data will be performed using an intention-to-treat analysis, all patients allocated to the control group or experimental group will be analysed. The descriptive analysis for each group at each assessment time will be expressed as number (percentage) for categorical variables, and as mean (SD) or Median [IQR] according to their normal or non-normal distribution for quantitative variables. As described above, the primary outcome for non-inferiority analysis is based on the comparison of the BMI at 1 year after inclusion between the two groups with a two-sided 95% confidence interval. Experimental strategy will be considered as non-inferior if the confidence interval upper limit of the difference in BMI between the 2 groups is less than 1 kg/m^2^ (non-inferiority margin) with a 0.025 alpha risk. The comparative analysis between the 2 groups for the primary or secondary criteria will be carried out using the appropriate tests: Chi^2^ test or Fisher's exact test for qualitative variables; Student's t test or non-parametric Wilcoxon test for quantitative variables, according to their normal or non-normal distribution. To determine prognosis factors associated with positive response to stepped-care model of DP after short IP, a regression model will be used to search for factors associated with the effect of the intervention, integrating the MRS score as the explained variable, and the CGI score, the HAQ11 and motivation questionnaire scores, and the severity of the weight deficit as explanatory variables.

#### Economic evaluation

A cost-utility (CUA) and cost-effectiveness (CEA) analyses will be carried out from the all-payers perspective (including statutory health insurance, complementary health insurances and patients’ out-of-pocket expenditures). In a secondary analysis, a societal perspective will be adopted to account for productivity losses in both treatment arms. The time horizon will be that of the follow-up (12 months) and as such in accordance with French guidelines, no discounting rate will be applied to costs and effectiveness [31].

The effectiveness criteria will be quality-adjusted life-years (QALYs) in the CUA and BMI in the CEA. QALY will be derived from patients’ responses to the SF-12 at inclusion, weight normalisation, 6 and 12 months. In the absence of a French utility value set for the SF-12, the British set will be used.

Only direct costs related to the pathology and incurred between inclusion and 12 months will be included in the main analysis (IP and DP, ambulatory consultations, complementary examinations, medical transport…). In the secondary analysis from the societal perspective, indirect costs due to parents’ productivity loss related to the pathology of their child will also be included. Data sources for costs will include questionnaires and the centre’s discharge database, which records all admissions (IP and DP).

Incremental cost-effectiveness ratios will be calculated by dividing the difference in mean costs in the two treatment arms by the difference in mean effectiveness. It will be expressed in cost per QALY gained at 12 months in the CUA and in cost per BMI point gained at 12 months in the CEA. Deterministic and probabilistic sensitivity analyses (bootstrapping methods) will be carried out to assess the uncertainty surrounding the results. Results will be placed on a cost-effectiveness plane and a cost-effectiveness acceptability curve will be constructed to determine the probability that the experimental strategy is cost-effective based on the decision-maker’s willingness-to-pay.

## Discussion

Treatment trials with anorexia nervosa patients are challenging [32], and even more in EOAN with very low prevalence and ambivalence to engage in treatment [1]. As a result, treatment trials for anorexia nervosa remain scarce [33]. COTIDEA trial will assess the non-inferiority of a stepped-care model of DP after of a short IP stabilisation versus a prolonged IP based on BMI at one-year follow-up after admission in two randomised groups of children aged from 8 to 13 years suffering from EOAN and initial severe undernutrition. It will also be the first to assess the cost-effectiveness of both strategies, emphasing the need of collaboration with economic analysts to optimize resource allocation in healthcare.

We hypothesize that DP could represent an alternative care modality with better acceptability and lower cost in the management of EOAN, compared to prolonged IP, but also better social adjustment in this specific pediatric population in line with recent studies in adolescents and young adults [8, 9].We strongly believe that relay DP management could represent an alternative care modality to shorten the duration of IP in EOAN.

COTIDEA was initiated before the COVID pandemic, but remains all the more relevant with the recent increase in incidence of eating disorders [34, 35] and justifies all the more the development and validation of alternative care modalities that would lead to a reduction in hospital length of stay and costs.

## Data Availability

Not applicable.

## Abbreviations

AP-HP: Assistance Publique Hôpitaux de Paris
BMI: Body mass index
CDI: Children depression inventory
CEA: Cost-Effectiveness analyses
CGI: Clinical global impression
CSQ-8: Customer satisfaction questionnaire
CUA: Cost-Utility analyses
DP: Day Patient treatment
EOAN: Early onset anorexia nervosa
HAQ-11S: Helping alliance questionnaire-11
IP: Inpatient treatment
MRS: Morgan and Russel scale
OP: Out Patient treatment
SF-12: Short-Form 12-item health survey
STAIC: State –Trait anxiety inventory for children
